# Cancer stem cells are prevalent in the basal-like 2 and mesenchymal triple-negative breast cancer subtypes *in vitro*

**DOI:** 10.3389/fcell.2023.1237673

**Published:** 2023-09-12

**Authors:** Maxim Olsson, Peter Larsson, Junko Johansson, Vasu R. Sah, Toshima Z. Parris

**Affiliations:** ^1^ Department of Chemistry and Molecular Biology, University of Gothenburg, Gothenburg, Sweden; ^2^ Department of Oncology, Institute of Clinical Sciences, Sahlgrenska Academy, University of Gothenburg, Gothenburg, Sweden; ^3^ Sahlgrenska Center for Cancer Research, Sahlgrenska Academy, University of Gothenburg, Gothenburg, Sweden; ^4^ Department of Surgery, Institute of Clinical Sciences, Sahlgrenska Academy, University of Gothenburg, Gothenburg, Sweden

**Keywords:** tumor-initiating cells, CD24, CD44, aldehyde dehydrogenase, flow cytometry

## Abstract

**Background:** Triple-negative breast cancer (TNBC) is an aggressive subtype with the most unfavorable clinical outcomes, in part due to tumor heterogeneity, treatment resistance, and tumor relapse. The TNBC subtypes [basal-like 1 (BL1), basal-like 2 (BL2), mesenchymal (M), and luminal androgen receptor (LAR)] are biologically and clinically distinct entities that respond differently to local and systemic therapies. Therefore, we need to have a better understanding of cancer stemness relating to drug-resistant populations in the TNBC subtypes.

**Methods:** Breast cancer stem cell (BCSC) distribution was investigated using an integrated flow cytometry approach with the ALDEFLUOR™ assay (ALDH) and CD24/CD44 antibodies. In total, 27 commercially available cell lines derived from normal and malignant mammary tissue were characterized into differentiated tumor cells and/or BCSC subpopulations (ALDH^−^CD44^+^CD24^-/low^ enriched mesenchymal-like BCSCs, ALDH^+^non-CD44^+^CD24^−/low^ enriched epithelial-like BCSCs, and highly purified ALDH^+^CD44^+^CD24^−/low^ BCSCs).

**Results:** BCSCs were not only enriched in estrogen receptor (ER) negative (mean, 49.6% *versus* 6.9% in ER+) and TNBC cell lines (51.3% *versus* 2.1% in Luminal A), but certain BCSC subpopulations (e.g., enriched mesenchymal-like BCSCs) were also significantly more common in the M (64.0% *versus* 6.2% in BL1; 64.0% *versus* 0% in LAR) and BL2 (77.4% *versus* 6.2% in BL1; 77.4% *versus* 0% in LAR; 77.4% *versus* 10.4% in TNBC UNS) TNBC subtypes. In contrast, ALDH status alone was not indicative of ER status or BC subtype.

**Conclusion:** Taken together, these findings demonstrate the enrichment of potentially treatment-resistant BCSC subpopulations in the M and BL2 triple-negative breast cancer subtypes.

## 1 Introduction

Triple-negative breast cancer (TNBC) is a heterogeneous disease comprised of four distinct molecular subtypes (basal-like 1 [BL1], basal-like 2 [BL2], mesenchymal [M], and luminal androgen receptor [LAR]) with differing biology (Lehmann et al., 2021), therapeutic vulnerability and response rates (Kyndi et al., 2008; Masuda et al., 2013; Lehmann et al., 2016; Speers et al., 2017), and recurrence rates (Zhang et al., 2021). During the past two decades, the systematic characterization of the intrinsic (tumor biology) and extrinsic features (tumor microenvironment) of TNBC using omics technologies has led to the development of novel therapeutic options, e.g., PARP inhibitors and immune-checkpoint inhibitors (Bianchini et al., 2022). Given the high tumor mutational burden (highest mutational levels in the BL1 and M subtypes) and immunogenic characteristics (the M subtype lacks immune cells and decreased antigen presentation) in most TNBCs, some patients with TNBC may benefit from immunotherapy with immune checkpoint blockade (Lehmann et al., 2021; Ribeiro et al., 2022). However, patients with TNBC have a 3-fold increased risk of early relapse within 3 years of diagnosis (Dent et al., 2007), in part due to intrinsic or acquired treatment resistance. Breast cancer stem cells (BCSCs) are tumor-initiating cells with invasive capacity that mediate metastasis, contribute to treatment resistance and cancer relapse (Liu et al., 2014; Li et al., 2017), and are prevalent in TNBCs (Charafe-Jauffret et al., 2009).

Three main BCSC subpopulations have been identified using the CD44^+^CD24^−/low^ phenotype and high aldehyde dehydrogenase (ALDH^+^) activity, i.e., ALDH^−^CD44^+^CD24^−/low^ enriched mesenchymal-like BCSCs (located along the tumor-invasive edge), ALDH^+^non-CD44^+^CD24^−/low^ enriched epithelial-like BCSCs (located centrally within a tumor), and highly purified ALDH^+^CD44^+^CD24^−/low^ BCSCs with both mesenchymal and epithelial characteristics are considered to be the most tumorigenic (Liu et al., 2014; Liu et al., 2018; Abdoli Shadbad et al., 2021). TNBCs with BCSC characteristics are associated with adverse clinical outcome, treatment resistance, tumor relapse, and aggressive tumor features (Abdoli Shadbad et al., 2021; Qiao et al., 2021). Nevertheless, it is still unclear whether BCSC distribution differs between the TNBC subtypes. Here, we characterize BCSC subpopulations in 27 breast cell lines (16 TNBC) derived from normal and malignant mammary tissue using an integrated flow cytometry approach with the ALDEFLUOR™ assay and CD24/CD44 antibodies.

## 2 Materials and methods

### 2.1 Cell lines

Twenty-four human breast cancer cell lines and a non-cancer cell line (MCF-10A) were purchased from the American Type Culture Collection (ATCC; Rockville, MD, United States) or Deutsche Sammlung von Mikroorganismen und Zellkulturen (Leibniz Institute DSMZ; Braunschweig, Germany). Göran Landberg and Julie Grantham (University of Gothenburg) kindly provided the MDA-MB-468 and MCF-7 cells, respectively. The cells were maintained in a humidified atmosphere containing 5% CO_2_ at 37°C and cultured in either RPMI 1640 or DMEM supplemented with 10% fetal bovine serum. MDA-MB-468 cells were supplemented with 1% sodium pyruvate, while BT-20 were supplemented with 1% Minimum Essential Medium Non-Essential Amino Acids (all ThermoFisher Scientific). The cell lines were classified into the breast cancer molecular subtypes (Luminal A, Luminal B, HER2 amplified [HER2amp], and TNBC) as previously described (Jiang et al., 2016; Guo et al., 2018; Gambardella et al., 2022). Cell lines characterized as TNBC were further stratified into the TNBCtype-4 molecular subtypes (BL1, BL2, M, and LAR) (Lehmann et al., 2016). Authentication of 11/27 cell lines was performed using the ATCC short tandem repeat profiling service or Eurofins Genomics Human Cell Line Authentication service. The 27 human breast cell lines are listed in Supplementary Table S1.

### 2.2 ALDEFLUOR™ and CD44^+^/CD24^−/low^ analysis

Flow cytometry was used to categorize each cell line into differentiated tumor cells (ALDH^-^non-CD44^+^CD24^−/low^), highly purified BCSCs (ALDH^+^CD44^+^CD24^−/low^), enriched epithelial-like BCSCs (ALDH^+^non-CD44^+^CD24^−/low^), and enriched mesenchymal-like BCSCs (ALDH^−^CD44^+^CD24^−/low^) according to the CD44^+^CD24^−/low^ phenotype and ALDH^br^ activity using the ALDEFLUOR™ Kit (Cat. 01700, STEMCELL Technologies, Cambridge, United Kingdom), PE-conjugated mouse anti-human CD24 antibodies (Cat. 555428, BD Biosciences), and APC-conjugated mouse anti-human CD44 antibodies (Cat. 559942, BD Biosciences). In brief, dry ALDEFLUOR™ reagent was suspended in DMSO, 2N HCl, and ALDEFLUOR™ Assay Buffer at room temperature. The fluorescent-activated ALDEFLUOR™ reagent was then aliquoted and stored at −20°C. Five cell lines (BT-549, HCC 1806, MCF-7, MDA-MB-453, and MDA-MB-468) were used to determine the optimal sample concentration (1 × 10^5^, 2 × 10^5^, and 5 × 10^5^ cells/mL) and ALDEFLUOR™ incubation time (15, 30, 45, and 60 min) at 37°C. The optimal sample concentration was determined by identifying the sample concentration with the largest difference in mean fluorescence intensity (MFI) between ALDH^br^ (ALDEFLUOR™) and ALDH^low^ (ALDEFLUOR™ + diethylaminobenzaldehyde [DEAB]) cells. The optimal sample concentration was 1 × 10^5^ cells/mL for all five cell lines, but the optimal incubation time was cell line-specific (15 min for BT-549, 30 min for MCF-7 and MDA-MB-453, 45 min for HCC1806, and 60 min for MDA-MB-468). For the remaining 22 cell lines, 1 × 10^5^ cells/mL and 40 min incubation time were used.

For each cell line, 2 × 10^5^ cells were resuspended in 1 mL ALDEFLUOR™ Assay Buffer and 5 µL activated ALDEFLUOR™ reagent. The cell suspension was mixed and 0.5 mL immediately transferred to another Eppendorf tube containing 5 µL ALDEFLUOR™ DEAB reagent. The test and DEAB control samples were then incubated at 37°C for the indicated incubation time. The control sample suspended in 400 µL ALDEFLUOR™ Assay Buffer was stored on ice while the test sample was stained with 90 µL ALDEFLUOR™ Assay Buffer containing 5 µL each of PE-CD24 and APC-CD44 antibodies for 30 min on ice, followed by 100 µL ALDEFLUOR™ Assay Buffer and 5 µL 7-AAD (Cat. 559925, BD Biosciences) for 10 min at room temperature in the dark. Each sample was resuspended in 300 µL ALDEFLUOR™ Assay Buffer and stored on ice in the dark until analysis. After filtering, a minimum of 10,000 events were analyzed using a BD Accuri™ C6 Personal Flow Cytometer (BD Biosciences, Franklin Lakes, New Jersey, United States) and FlowJo™ v10.8.1 Software (BD Life Sciences). A minimum of two biological and technical replicates were performed on selected cell lines. The gating strategy is outlined in Supplementary Figure S1.

### 2.3 Statistical analysis

The statistical analyses were performed using a 0.05 *p*-value cutoff in R/Bioconductor version 4.0.4. Values are presented as the mean and standard deviation (SD). The Shapiro-Wilks normality test was performed to determine whether the parametric T-test (normally distributed, *p* > 0.05) or non-parametric Wilcoxon test (not normally distributed, *p* < 0.05) should be used. Plots were constructed using the moonBook (version 0.3.1) (Moon, 2015), ggplot2 (version 3.3.6) (Kassambara, 2019), and ggpubr (version 0.4.0) (Kassambara et al., 2019) packages in R.

## 3 Results

### 3.1 BCSC populations are enriched in BL2 and M triple-negative breast cancer cell lines

**FIGURE 1:**
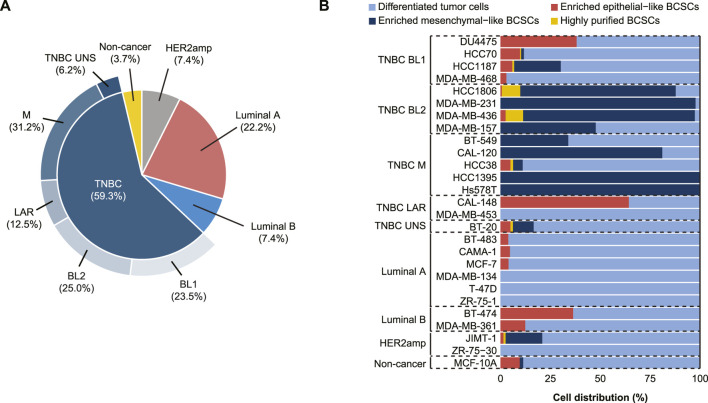
Characterization of 27 cell lines derived from normal and malignant mammary tissue into breast cancer stem cell (BCSC) subpopulations. **(A)** Breast cancer subtyping (Luminal A, Luminal B, HER2amp, and TNBC) and TNBCtype-4 subtypes [basal-like 1 (BL1), basal-like 2 (BL2), mesenchymal (M), luminal androgen receptor (LAR), and unspecified (UNS)] for the 27 cell lines. **(B)** BCSC classification using flow cytometry analysis with antibodies for CD24, CD44, and ALDEFLUOR™ demonstrates that cell lines representing the TNBC subtypes (BL1, BL2, M, LAR, and UNS) contain different BCSC populations, whereas luminal ER+ cells primarily contain differentiated tumor cells.

In total, 16/27 human breast cell lines included in the study were TNBC (59.3%), of which 23.5% were characterized as BL1, 25.0% as BL2, 12.5% as LAR, 31.2% as M, and 6.2% as TNBC unspecified (Figure 1A). BCSC populations were more prevalent in TNBC, while differentiated tumor cells (ALDH^-^non-CD44^+^CD24^-/low^) were primarily found in Luminal A and HER2amp cells (Figure 1B, Supplementary Figure S2). In TNBC cell lines, enriched mesenchymal-like BCSCs (ALDH^−^CD44^+^CD24^−/low^) were predominantly identified in the BL2 and M subtypes, while enriched epithelial-like BCSCs (ALDH^+^non-CD44^+^CD24^−/low^) were found in 16/27 (59.3%) cell lines. Although rare, highly purified BCSCs (ALDH^+^CD44^+^CD24^−/low^) were only identified in one HER2amp cell line (JIMT-1) and TNBC cell lines, particularly HCC1806 and MDA-MB-436 cells (both BL2 subtype). These findings demonstrate the existence of distinct BCSC subpopulations based on TNBC subtype.

**FIGURE 2:**
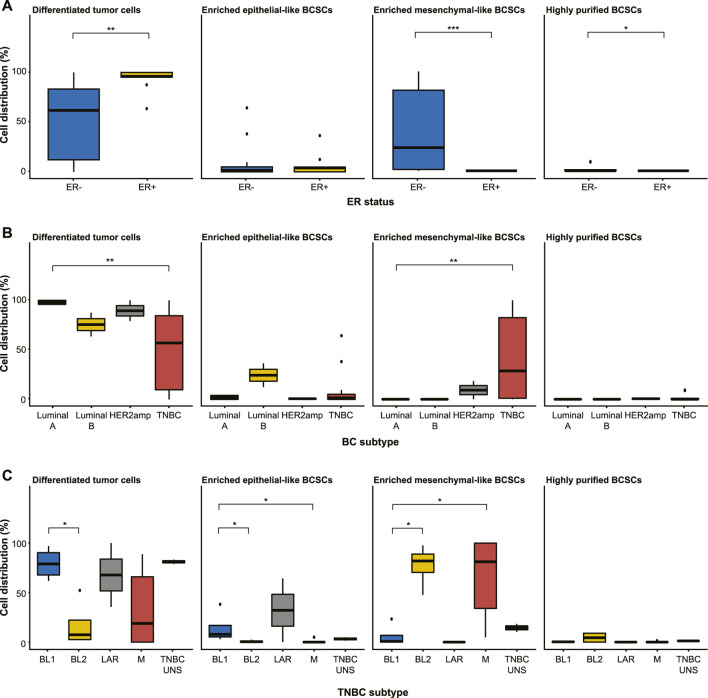
Distribution of differentiated tumor cells and BCSC subpopulations (enriched epithelial-like BCSCs, enriched mesenchymal-like BCSCs, and highly purified BCSCs) in the 27 cell lines stratified by **(A)** estrogen receptor (ER) status, **(B)** breast cancer subtyping (Luminal A, Luminal B, HER2amp, and TNBC), and **(C)** TNBCtype-4 subtypes [basal-like 1 (BL1), basal-like 2 (BL2), mesenchymal (M), luminal androgen receptor (LAR), and unspecified (UNS)]. Error bars depict the standard deviation. *p*-values calculated using Wilcoxon test. ns = not significant (*p* > 0.05); *p ≤ 0.05; **p ≤ 0.01; ***p ≤ 0.001; ****p ≤ 0.0001.

Further examination of the distribution of differentiated tumor cells and BCSC subpopulations in the 27 cell lines revealed no significant differences in enriched epithelial-like BCSCs based on estrogen receptor (ER) status or BC subtype (*p* > 0.05; Figure 2; Table 1). However, significantly more enriched epithelial-like BCSCs were found in BL1 cells than BL2 and M cells (mean ± SD, 14.2% ± 16.2 *versus* 0.8% ± 1.2 in BL2 and 1.0% ± 2.2 in M). Differentiated tumor cells were significantly more common in ER+ (93.1% ± 11.8 *versus* 50.5% ± 37.6 in ER-), Luminal A (97.9% ± 2.4 *versus* 48.7% ± 38.0 in TNBC), and BL1 TNBC (79.2% ± 16.2 *versus* 17.3% ± 23.7 in BL2; *p* < 0.05). In contrast, enriched mesenchymal-like BCSCs were predominantly found in ER- (40.2% ± 40.8 *versus* 0% ± 0 in ER+), TNBC (41.5% ± 41.7 *versus* 9.2% ± 13.0 in Luminal A), as well as BL2 (77.4% ± 21.4 *versus* 6.2% ± 11.5 in BL1) and M TNBC cells (64.0% ± 42.7 *versus* 6.2% ± 11.5 in BL1). Although 2/4 BL2 cell lines showed the highest distribution of highly purified BCSCs (9.3% in HCC1806 and 8.8% in MDA-MB-436 cells), significantly more highly purified BCSCs were generally found in ER- (1.4% ± 2.9 *versus* 0% ± 0 in ER+; *p* < 0.05).

**TABLE 1:** Distribution (in percent) of differentiated tumor cells and BCSC subpopulations in human breast cell lines.

	Differentiated tumor cells	Enriched epithelial-like BCSCs	Enriched mesenchymal-like BCSCs	Highly purified BCSCs
ER status				
ER+	93.1 ± 11.8	6.9 ± 11.8	0	0
ER-	50.5 ± 37.6	8.0 ± 17.2	40.2 ± 40.8	1.4 ± 2.9
BC subtype				
HER2amp	89.5 ± 14.8	0.7 ± 1.0	9.2 ± 13.0	0.6 ± 0.8
Luminal A	97.9 ± 2.4	2.1 ± 2.4	0	0
Luminal B	75.6 ± 17.0	24.4 ± 17.0	0	0
TNBC	48.7 ± 38.0	8.4 ± 17.7	41.5 ± 41.7	1.4 ± 3.0
TNBC subtype				
BL1	79.2 ± 16.2	14.2 ± 16.2	6.2 ± 11.5	0.4 ± 0.5
BL2	17.3 ± 23.7	0.8 ± 1.2	77.4 ± 21.4	4.5 ± 5.2
LAR	67.8 ± 45.5	32.2 ± 45.5	0	0
M	34.7 ± 40.5	1.0 ± 2.2	64.0 ± 42.7	0.3 ± 0.6
TNBC UNS	83.3	5	10.4	1.3

Note: Data shown as mean ± SD (standard deviation) BC, breast cancer; BCSC, breast cancer stem cell; BL1, basal-like 1; BL2, basal-like 2; ER, estrogen receptor; LAR, luminal androgen receptor; M, mesenchymal; TNBC, triple-negative breast cancer; UNS, unspecified.

### 3.2 The ALDEFLUOR™ assay alone does not distinguish between the BC subtypes

**FIGURE 3:**
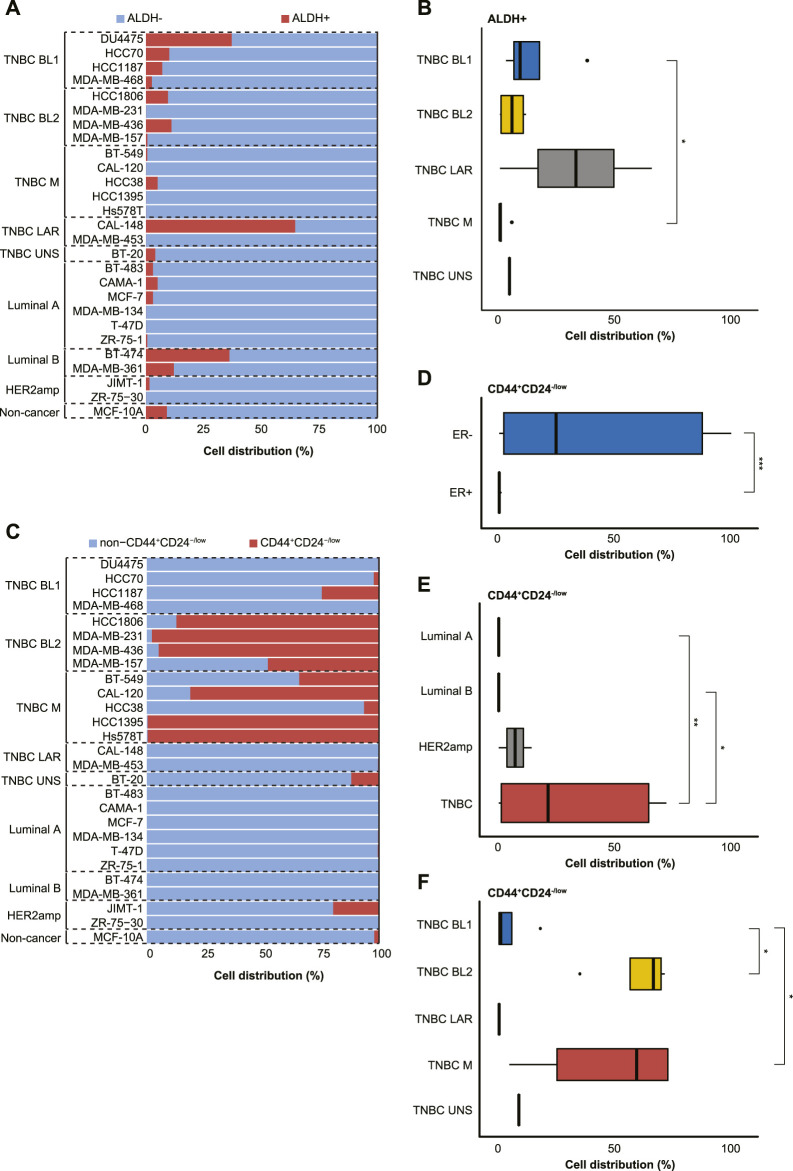
Characterization of 27 cell lines derived from normal and malignant mammary tissue into breast cancer stem cell (BCSC) subpopulations using the **(A,B)** ALDEFLUOR™ assay (ALDH+ or ALDH-) or **(C–F)** CD44^+^/CD24^−/low^ phenotype (CD44^+^/CD24^−/low^ or non-CD44^+^/CD24^−/low^). The cell lines were stratified by estrogen receptor (ER) status, breast cancer subtyping (Luminal A, Luminal B, HER2amp, and TNBC) or TNBCtype-4 subtypes [basal-like 1 (BL1), basal-like 2 (BL2), mesenchymal (M), luminal androgen receptor (LAR), and unspecified (UNS)] for the 27 cell lines. Error bars depict the standard deviation. *p*-values calculated using Wilcoxon test. ns = not significant (*p* > 0.05); *p ≤ 0.05; **p ≤ 0.01; ***p ≤ 0.001; ****p ≤ 0.0001.

The distribution of ALDH^br^ cells (ALDH+ and ALDH-) and the CD44^+^/CD24^−/low^ phenotype (CD44^+^CD24^−/low^ and non-CD44^+^CD24^−/low^) were then analyzed separately, thereby demonstrating that ALDH-negativity was prevalent in the majority of cell lines (Figure 3A). Although no clear difference was found in ALDH status based on ER status or BC subtype, all 4 BL1 cell lines and 4/6 Luminal A cell lines contained a small subpopulation of ALDH+ cells. Furthermore, a significantly higher proportion of BL1 cells (14.1% ± 15.6) were ALDH+ than mesenchymal-like TNBC cells (1.1% ± 2.2; *p* < 0.05; Figure 3B). Intriguingly, only three cell lines (i.e., BT-474 [Luminal B], DU4475 [BL1], and CAL-148 [LAR]) had >30% ALDH+ cells. With the exception of the LAR TNBC subtype, all other TNBC subtypes contained CD44^+^CD24^−/low^ subpopulations, while Luminal A and Luminal B only contained non-CD44^+^CD24^−/low^ subpopulations (Figure 3C). Moreover, the CD44^+^/CD24^−/low^ phenotype was associated with ER-negativity (41.5% ± 41.7 *versus* 0.06% ± 0.09 in ER+), TNBC status (42.9% ± 42.7 *versus* 0.08% ± 0.1 in Luminal A and 0.008% ± 0.01 in Luminal B), BL2 (81.9% ± 23.2 *versus* 6.6% ± 11.9 in BL1) and M TNBC cells (64.2% ± 42.0 *versus* 6.6% ± 11.9 in BL1; *p* < 0.05; Figures 3D–F).

## 4 Discussion

Breast cancer stem cells have previously been shown to be enriched in estrogen receptor-negative and triple-negative breast cancer (Charafe-Jauffret et al., 2009; Liu et al., 2018). This is however, to our knowledge, the first study to report the prevalence of BCSC subpopulations in cell lines representing different TNBCtype-4 molecular subtypes using an integrated flow cytometry approach combining the CD44^+^/CD24^−/low^ phenotype and ALDEFLUOR™ assay. BCSCs were indeed more prevalent in ER- and TNBC cells, while differentiated tumor cells (ALDH^-^non-CD44^+^CD24^−/low^) were primarily found in Luminal A and HER2amp cells. Intriguingly, mesenchymal-like BCSCs were predominantly found in cell lines derived from ER- breast cancer and the M and BL2 TNBC subtypes, while epithelial-like BCSCs were detected in the BL1 and LAR TNBC subtypes. Used as separate BCSC biomarkers, the CD44^+^/CD24^−/low^ phenotype was also more indicative of ER, BC and TNBC status than ALDH activity.

To our knowledge, only one other study has investigated BCSC distribution using this integrated approach in a single experiment (Liu et al., 2018). However, Liu *et al.* only studied two TNBC patient-derived xenografts without consideration of their TNBC subtype. Other studies either used one BCSC marker (CD44^+^/CD24^−/low^ or aldehyde dehydrogenase expression) or both in two separate experiments (Sheridan et al., 2006; Jaggupilli and Elkord, 2012; Li et al., 2017; Qiao et al., 2021). Integration of both BCSC biomarkers allowed us to identify differentiated tumor cells and three BCSC subpopulations, thereby revealing distinct differences in BCSC distribution depending on ER status, as well as BC and TNBC subtyping. Although the CD44^+^/CD24^−/low^ phenotype was also a good indicator of BCSC distribution in the BL2 and M TNBC subtypes, we would not have been able to identify highly purified BCSCs (ALDH^+^CD44^+^CD24^−/low^) without including the ALDEFLUOR™ assay.

BCSCs are often associated with metastatic spread and treatment resistance. Here, we show that the BL2 and M TNBC subtypes are primarily comprised of enriched mesenchymal-like BCSCs. Lehmann *et al.* recently illustrated that the BL2 and M subtypes may be derived from myoepithelial/basal cells, though the origin of the M subtype points to de-differentiated BL1 cells (Lehmann et al., 2021). Molecular profiling also showed that the M subtype expresses adhesion and motility genes consistent with epithelial-mesenchymal transitioning in mesenchymal-like BCSCs, while the BL2 subtype expresses genes linked to DNA repair and development (Lehmann et al., 2021). BL2 was also less likely to achieve pathologic complete response following neoadjuvant treatment and had the worst relapse-free survival (Lehmann et al., 2016).

The distribution of ALDH+ cells in cell lines generally varies between studies, e.g., ALDH-positivity varies from 2.6% to 48% for the HCT116 cell line (Deng et al., 2010; Muraro et al., 2012; Morita et al., 2013; Suzuki et al., 2013). In the present study, we only show three cell lines with >30% ALDH+ cells (i.e., BT-474, DU4475, and CAL-148), which is in contrast to a study by Charafe-Jauffret *et al.* (Charafe-Jauffret et al., 2009) showing breast cancer cells with 5%–99% or 100% ALDH-positivity (e.g., HCC38 *versus* 5% in the current study) and 100% ALDH-positivity for BT-474 (36% in the current study) by Zhou *et al.* (Zhou et al., 2019). These discrepancies might be due to differences in seeding density (Opdenaker et al., 2015), sample concentration (cells/mL), and ALDEFLUOR™ incubation time, which are known to influence the fluorescence intensity of ALDH^br^ cells. Although STEMCELL Technologies does not specify to optimize the seeding density, Opdenaker and colleagues observed fewer ALDH+ cells and decreased expression of ALDH isoforms in cancer cell lines grown at high density (Opdenaker et al., 2015). As a general rule of thumb, the cell cultures were never grown beyond 70% confluency in the present study. However, optimization of the ALDEFLUOR™ assay was only performed for 5/27 cell lines (BT-549, HCC1806, MCF-7, MDA-MB-453, and MDA-MB-468) included in the study. This should have ideally been done for all 27 cell lines to ensure more accurate and reproducible results at the optimal assay incubation time and sample concentration.

Taken together, flow cytometry analysis of breast cancer cell lines revealed distinct patterns of BCSC biomarker expression based on ER status and subtyping (BC and TNBC). BCSC subpopulations were more prevalent in cell lines derived from ER-negative breast cancer, as well as the M and BL2 TNBC subtypes. However, further evaluation of BCSC distribution is warranted in TNBC patient biopsies (classified by the TNBCtype-4 molecular subtypes) in relation to treatment response and clinical outcome.

## Data Availability

The original contributions presented in the study are included in the article/Supplementary Material, further inquiries can be directed to the corresponding author.
