# Supplementary material for: Cancer stem cells are prevalent in the basal-like 2 and mesenchymal triple-negative breast cancer subtypes in vitro
Source: Front Cell Dev Biol. 2023 Sep 12;11:1237673. doi: 10.3389/fcell.2023.1237673 (PMC10523387; doi:10.3389/fcell.2023.1237673)
Supplement: Supplementary file 1 [file DataSheet1.pdf]

frontiers

Supplementary Material

Cancer stem cells are prevalent in the basal-like 2 and mesenchymal-like triple-negative breast cancer subtypes *in vitro*

Maxim Olsson, Peter Larsson, Junko Johansson, Vasu R. Sah, and Toshima Z. Parris*

*** Correspondence:** Toshima Z. Parris: toshima.parris@oncology.gu.se

1 Supplementary Figures

2 Supplementary Tables

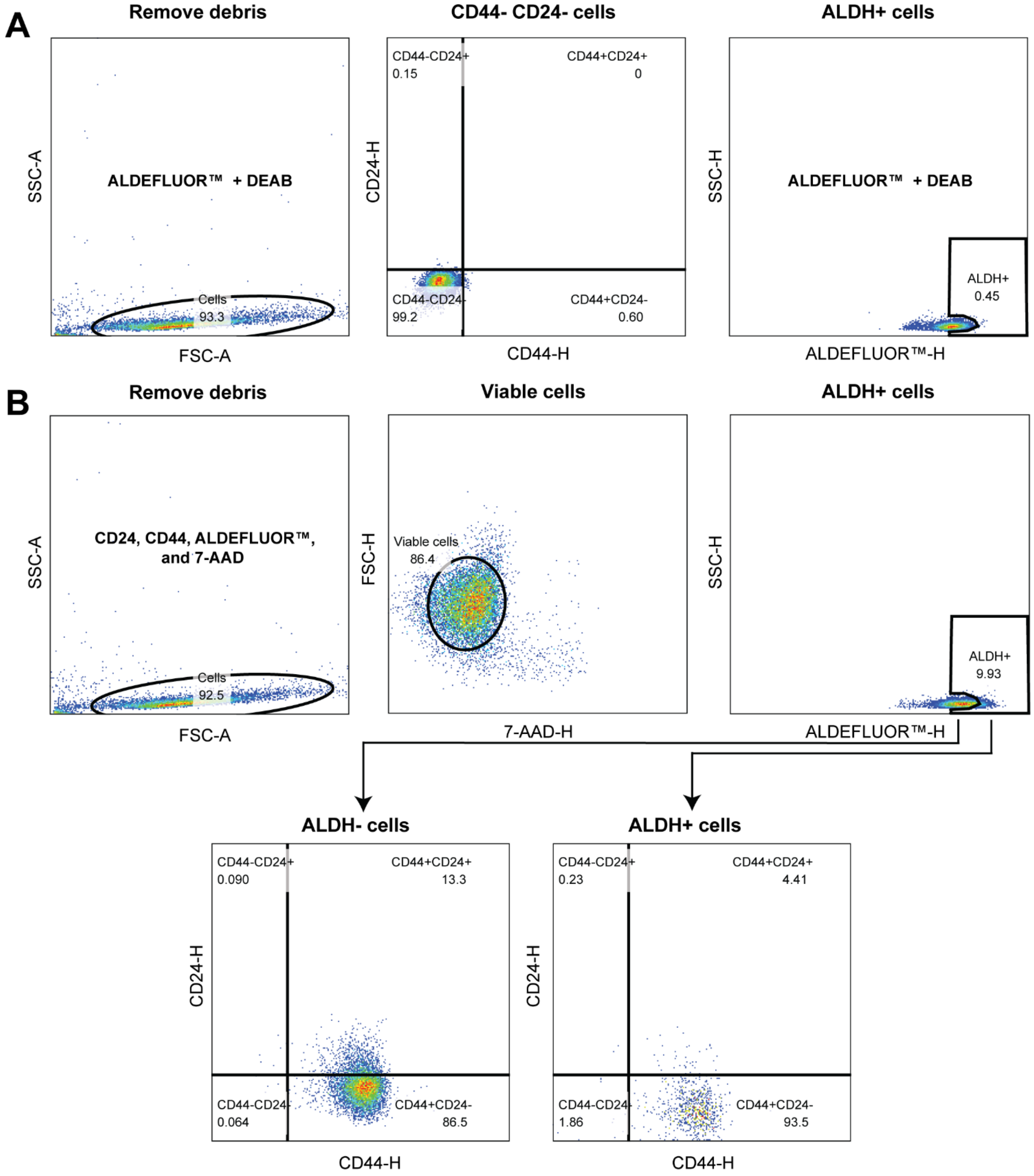

Supplementary Figure S1. Gating strategy for flow cytometry analysis of breast cancer stem cell sub-populations using the ALDEFLUOR™ assay, CD44, and CD24. All samples were gated to remove debris and cell doublets, and to only include viable cells using 7-AAD (7-Aminoactinomycin D). (**A**) According to the manufacturer's instructions, a sample containing ALDEFLUOR™ DEAB reagent was used to account for background fluorescence in the ALDEFLUOR™ assay, in combination with fluorescence minus one (FMO) controls to determine the negative gates. (**B**) Breast cancer stem cell

sub-populations were then gated using the negative controls to identify $ALDH^{br}$/$ALDH^{low}$ cells in combination with CD44 and CD24 expression.

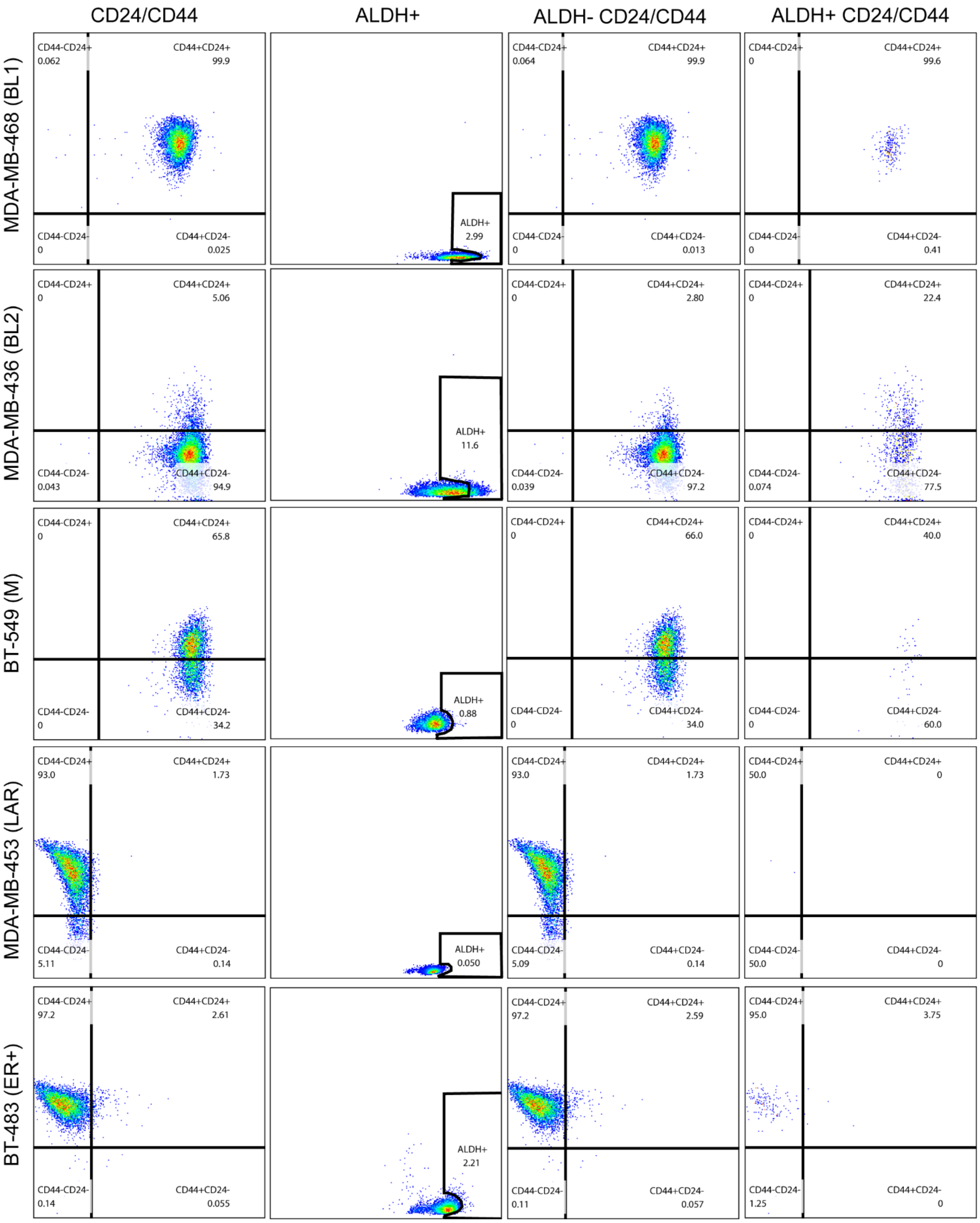
CD24/CD44
ALDH+
ALDH- CD24/CD44
ALDH+ CD24/CD44
MDA-MB-468 (BL1)
MDA-MB-436 (BL2)
BT-549 (M)
MDA-MB-453 (LAR)
BT-483 (ER+)
CD44-CD24+ 0.062
CD44+CD24+ 99.9
CD44-CD24- 0
CD44+CD24- 0.025
ALDH+ 2.99
CD44-CD24+ 0.064
CD44+CD24+ 99.9
CD44-CD24- 0
CD44+CD24- 0.013
CD44-CD24+ 0
CD44+CD24+ 99.6
CD44-CD24- 0
CD44+CD24- 0.41
CD44-CD24+ 0
CD44+CD24+ 5.06
CD44-CD24- 0.043
CD44+CD24- 94.9
ALDH+ 11.6
CD44-CD24+ 0
CD44+CD24+ 2.80
CD44-CD24- 0.039
CD44+CD24- 97.2
CD44-CD24+ 0
CD44+CD24+ 22.4
CD44-CD24- 0.074
CD44+CD24- 77.5
CD44-CD24+ 0
CD44+CD24+ 65.8
CD44-CD24- 0
CD44+CD24- 34.2
ALDH+ 0.88
CD44-CD24+ 0
CD44+CD24+ 66.0
CD44-CD24- 0
CD44+CD24- 34.0
CD44-CD24+ 0
CD44+CD24+ 40.0
CD44-CD24- 0
CD44+CD24- 60.0
CD44-CD24+ 93.0
CD44+CD24+ 1.73
CD44-CD24- 5.11
CD44+CD24- 0.14
ALDH+ 0.050
CD44-CD24+ 93.0
CD44+CD24+ 1.73
CD44-CD24- 5.09
CD44+CD24- 0.14
CD44-CD24+ 50.0
CD44+CD24+ 0
CD44-CD24- 50.0
CD44+CD24- 0
CD44-CD24+ 97.2
CD44+CD24+ 2.61
CD44-CD24- 0.14
CD44+CD24- 0.055
ALDH+ 2.21
CD44-CD24+ 97.2
CD44+CD24+ 2.59
CD44-CD24- 0.11
CD44+CD24- 0.057
CD44-CD24+ 95.0
CD44+CD24+ 3.75
CD44-CD24- 1.25
CD44+CD24- 0

Supplementary Figure S2. Representative flow cytometry dot plots of breast cancer stem cell sub-populations using the ALDEFLUOR™ assay, CD44, and CD24. One cell line per breast cancer subtype (TNBC BL1, TNBC BL2, TNBC M, TNBC LAR, and ER+) are shown.

Supplementary Table 1. Cell lines included in the study derived from breast cancer and normal breast tissue

	Cell line	Breast cancer subtype[A]	TNBC subtype[A]	Site of origin[B]	Culture medium	Authenticated
1	BT-20	TNBC	TNBC UNS[a]	PT	DMEM + 10% FBS + 1% NEAA	
2	BT-474	Luminal B[b]	-	PT	DMEM + 10% FBS	Eurofins
3	BT-483	Luminal A[b]	-	PT	RPMI + 10% FBS	
4	BT-549	TNBC	M[a]	PT	RPMI + 10% FBS	Eurofins
5	CAL-120	TNBC	M[a]	M	DMEM + 10% FBS	
6	CAL-148	TNBC	LAR[a]	M (PE)	DMEM + 10% FBS	Eurofins
7	CAMA-1	Luminal A[b]	-	M	DMEM + 10% FBS	
8	DU4475	TNBC	BL1[a]	M	RPMI + 10% FBS	
9	HCC1187	TNBC	BL1[a]	PT	RPMI + 10% FBS	
10	HCC1395	TNBC	M[a]	PT	RPMI + 10% FBS	
11	HCC1806	TNBC	BL2[a]	PT	RPMI + 10% FBS	Eurofins
12	HCC38	TNBC	M[a]	PT	RPMI + 10% FBS	ATCC
13	HCC70	TNBC	BL1[a]	PT	RPMI + 10% FBS	Eurofins
14	Hs578T	TNBC	M[a]	PT	DMEM + 10% FBS	
15	JIMT1	HER2amp[b]	-	M	DMEM + 10% FBS	
16	MCF-10A	Non-cancer	-	F	RPMI + 10% FBS + 0.5 mg/ml HC + 20 ng/mL EGF + 100 ng/ml CT + 10 µg/mL insulin	
17	MCF-7	Luminal A[b]	-	M	DMEM + 10% FBS	ATCC
18	MDA-MB-134-VI	Luminal A[c]	-	M	DMEM + 10% FBS	
19	MDA-MB-157	TNBC	BL2[a]	M (PE)	DMEM + 10% FBS	
20	MDA-MB-231	TNBC	BL2[a]	M (PE)	DMEM + 10% FBS	
21	MDA-MB-361	Luminal B[b]	-	M	DMEM + 10% FBS	
22	MDA-MB-436	TNBC	BL2[a]	M (PE)	DMEM + 10% FBS	ATCC

23	MDA-MB-453	TNBC	LAR[a]	M (PE)	DMEM + 10% FBS	Eurofins
24	MDA-MB-468	TNBC	BL1[a]	M (PE)	RPMI + 10% FBS + 1% SP	Eurofins
25	T47D	Luminal A[b]	-	M	DMEM + 10% FBS	
26	ZR-75-1	Luminal A[b]	-	M	DMEM + 10% FBS	
27	ZR-75-30	HER2amp[c]	-	M	RPMI + 10% FBS	ATCC/Eurofins

BL1, basal-like 1; BL2, basal-like 2; CT, cholera toxin; EGF, epidermal growth factor; FBS, fetal bovine serum; HC, hydrocortisone; LAR, luminal androgen receptor; M, mesenchymal; NEAA, non-essential amino acids; SP, sodium pyruvate; TNBC, triple-negative; UNS, unspecified

[A]Subtype classification according to [a]Guo, Y., et al. (2018), doi: 10.3390/genes9010029; [b]Gambardella, G., et al. (2022), doi: 10.1038/s41467-022-29358-6; cJiang, G., et al. (2016), doi: 10.1186/s12864-016-2911-z.

[B]Site of origin: PT, primary tumor; M, metastasis; PE, pleural effusion; F, fibrocystic disease
